# Continuous suture closure using a LapraTy® suture clips is an effective method for reconstruction of cystic duct stump after laparoscopic subtotal cholecystectomy

**DOI:** 10.1016/j.heliyon.2023.e20043

**Published:** 2023-09-21

**Authors:** Mitsugi Shimoda, Yu Kuboyama, Shuji Suzuki

**Affiliations:** Tokyo Medical University, Ibaraki Medical Center, Department of Gastroenterological Surgery, Japan

## Abstract

**Objective:**

Recently, number of laparoscopic subtotal cholecystectomy (LSC) has been increasing.

**Summary background data:**

LSC is suitable as a treatment as it can avoid intraoperative bile duct injury and bleeding for difficult laparoscopic cholecystectomy. On the other hand, improper handling of remnant of GB can lead to postoperative bile leakage.

**Methods:**

Here, we report our positive experience utilizing new technique of continuous suture closure and omental covering using Lapra Ty® suture clips on the remnant of GB.

**Results:**

From January 2016 to July 2021, we experienced 30 cases of LSC for LC patients who had difficulty securing critical view of safety (CVS). In six of the 30 cases, we repaired remnant of GB using continuous suture closure and omental covering with Lapra Ty® suture clips. The median operating time was 136 min (range 112–199 ml), and amount of bleeding was 1 ml (range 1–100). There were no cases of postoperative bile leakage (postope. BL), remnant cystic duct stone, and abscess formation in abdomen.

**Conclusion:**

we recommend this new suturing technique for closure of remnant of GB as it was very effective in preventing postope. BL after LSC.

## 1 Introduction

Recently, the number of patients undergoing laparoscopic cholecystectomy (LSC) has been on increase and has become even more common [[1], [2], [3], [4]]. Such as in patients with severe cholecystitis where laparoscopic total cholecystectomy (LTC) is unapplicable, LSC is suitable as it can avoid intraoperative bile duct injury and bleeding. However, the remnant of GB needs to be sutured after LSC. In such cases, Strasberg recommends conducting fenestration or reconstituting techniques for cystic duct suturing [5]. Both procedures are difficult to conduct under laparoscopy, and requires expertise technique and experience, and both techniques under laparoscopy pose a high risk of postoperative bile leakage (postope. BL) due to wall damage caused by too strong and/or inadequate ligation.

Here, we report a positive experience utilizing new technique of continuous suture closure and omental covering using Lapra Ty® suture clips on the remnant of GB.

## 2 Material and methods

From April 2019 to July 2021, we experienced 6 cases of LSC for difficulty LTC, and treated remnant of GB using continuous suture closure and omental covering with Lapra Ty® suture clips.

As this is a retrospective non-intervention study, the patients who completed follow-ups were also included in the patient group. If GB cancer is suspected pre- or intra-operatively, LSC was contraindicated. All procedures performed in studies involving human participants were in accordance with the ethical standards of the institutional research committee and with the 1995 Helsinki Declaration.

### 2.1 Surgical procedure

Our surgical procedure for LSC shown as below.

**Fig. 1:**
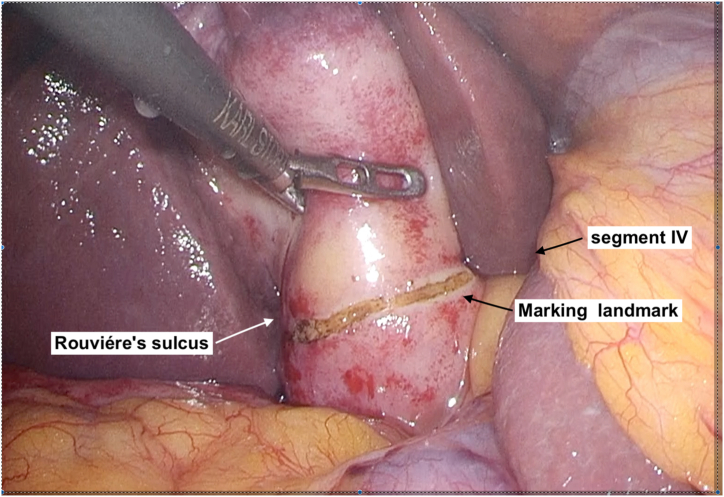
At first, we marked of superficial landmarks such as Rouviére's sulcus and segment IV of the liver, the infundibulum of the gallbladder using monopolar electrocautery.

**Fig. 2:**
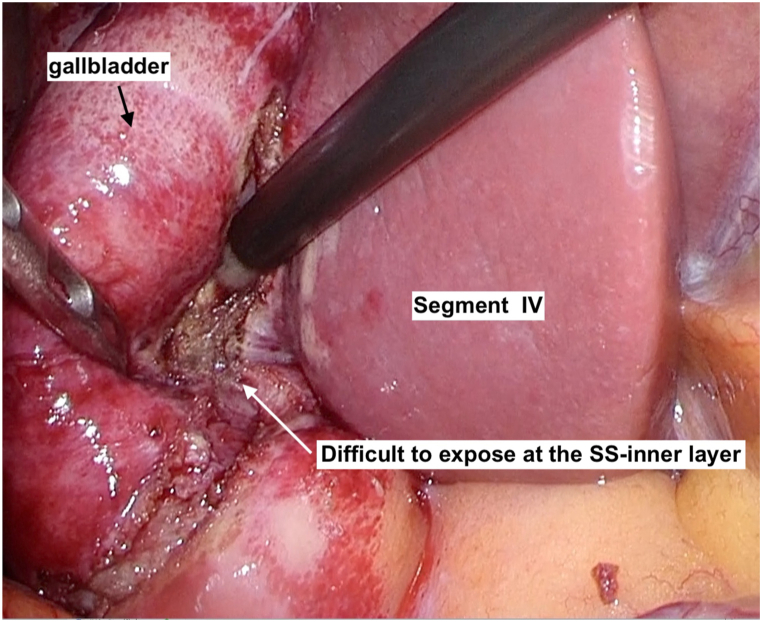
Next, we tried to expose CVS, but was unable to due to severe inflammation. We had to decide converse to subtotal cholecystectomy under laparoscopy.

LSC was performed using the standard four-ports two handed technique in the American position. At first, we confirmed and marked superficial landmarks such as Rouviére's sulcus, segment IV of the liver, and the infundibulum of the gallbladder (GB), using monopolar electrocautery (Fig. 1) [6]. Critical-View of Safety (CVS) was performed to expose the GB, and have been utilized as a valuable element to safely implement total cholecystectomy [7]. If we were unable to conduct CVS due to severe inflammation, we selected either of the two options: 1) the procedure was converted from open surgery to subtotal cholecystectomy, or 2) subtotal cholecystectomy was continuously performed under laparoscopy (Fig. 2). The attending surgeons were responsible to decide which options to proceed.

**Fig. 3:**
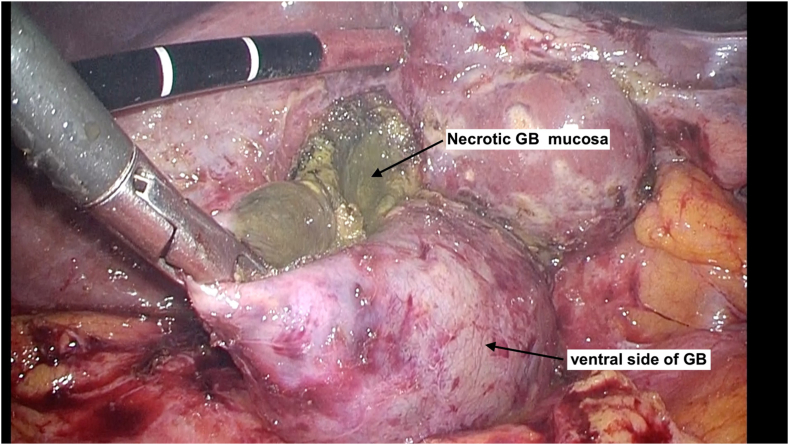
Then we underwent dome down procedure from the bottom of the GB, and if it was difficult to expose at the SS-inner layer, we immediately opened the ventral side of GB wall.

**Fig. 4:**
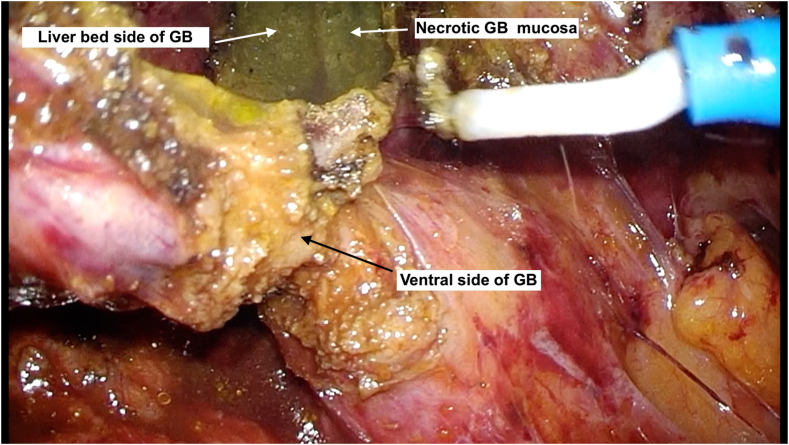
We carefully continued to resect ventral side of GB wall.

**Fig. 5:**
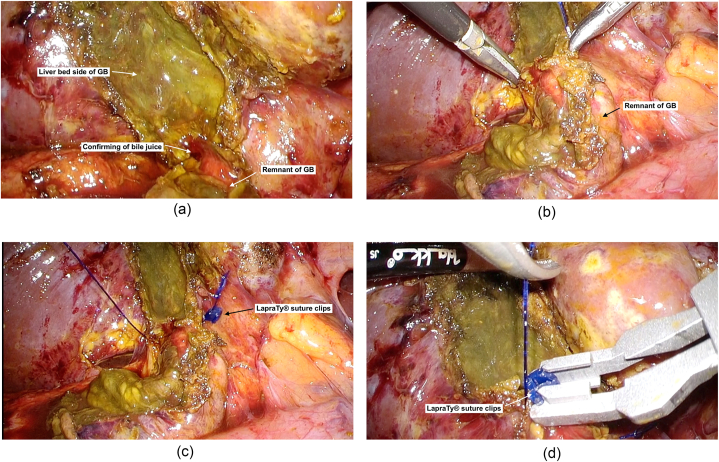
a–d. Finally, we conducted subtotal cholecystectomy, once confirming outflow of bile juice from remnant of GB (a). Then, we started continuous suture from the liver side (b). and both right and left sides were ligated with LapraTy® suture clips (Ethicon, Johnson and Johnson, Arlington, TX, USA, c and d).

Upon selecting option 2, the ventral side of GB wall was opened (Fig. 3) and resection continued to GB wall, and ventral and liver bed side (Fig. 4). Finally, to prevent residual gallstones and cholecystitis, we confirmed the outflow of bile juice from remnant GB (Fig. 5a), and in case the outflow was detected, we incinerated the gallbladder mucosa.

After LSC, in cases where the remnant wall of GB was too fragile or the stapler could not be applied sufficiently, we selected continuous suture closure of remnant of GB using LapraTy® suture clips (Ethicon, Johnson and Johnson, Arlington, TX, USA). We started continuous suture from the liver side (Fig. 5b), and both right and left sides were ligated with LapraTy® suture clips (Fig. 5 c and d).

**Fig. 6:**
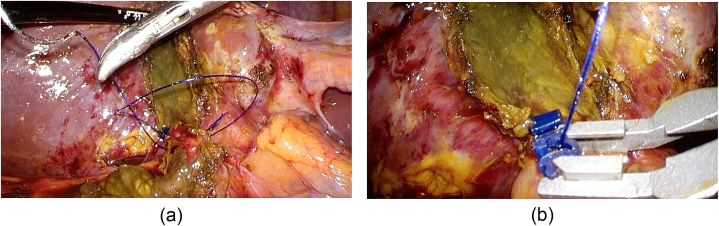
The second suture was performed in the same manner as first stich (a), using LapraTy® on the right side only (b).

**Fig. 7:**
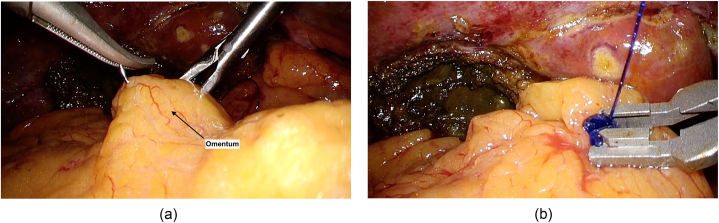
Omentum was also sutured (a) and secured in the same manner with LapraTy® to complete the covering to the remnant of GB (b).

The second suture was performed in the same manner as first stich, using LapraTy® on the right side only (Fig. 6 a and b). After 2–3 sutures, omentum was also sutured and secured in the same manner with LapraTy® to complete the covering to the remnant of GB (Fig. 7 a and b).

After suturing the remnant of GB, we placed closed drain to the foramen of Winslow, and measurements of bilirubin level from drainage tube was taken at first postoperative day (POD). If the bile leakage was not identified, we removed the drainage tube at 3POD. If bile leakage was suspected, basically we considered endoscopic biliary drainage or reoperation under the patient's condition. After one month, the patient was followed up in the outpatient clinic, and evaluated for postoperative biliary complication using MRCP imaging. We were unable to obtain evaluation from 3 of the patients as they refused MRCP.

## 3 Results

The treatment group constituted of 2 female and 4 male, median age was 65.5 years old (range 43–79 years old), and postoperative days was 1 day (range 1–13 days).

All of six cases had undergone LSC after at least 72 h of conservative treatment. Preoperative median white cell counts was 8250 μl（6000–12400 μl）, C-reactive protein (CRP) was 0.095 mg/dl（0.03–3.53 mg/dl）. One of the six case had grade I and 5 of six cases had grade II in acute cholecystitis classification. Two cases had preoperative percutaneous transhepatic GB drainage (PTGBD), and 3 cases had percutaneous transhepatic GB aspiration (PTGBA). Three cases underwent endoscopic lithotripsy before operation (Table 1). On preoperative MRCP findings, the cystic duct was not detected in 3 cases, and GB was not identified in 1 case. Median operating time was 136 min (range 112–199) and amount of bleeding was 1 ml (range 1–100 ml). There were no cases of postope. BL, remnant cystic duct stone and abscess formation in abdomen. We also evaluated postoperative biliary complication using MRCP imaging, and there were no cases of biliary related complication such as biliary stenosis, remnant cystic duct stone, and we did not have any cases of remnant cholecystitis（Table 2）.

**Table 1:** Patient's preoperative features.

case	Age (year)	gender	WBC (/μL)	CRP (mg/dL)	AC Grade	PTGBD (yes/no)	PTGBA yes/n)	EL (yes/n	Cystic duct^a^ (yes/n)	GB^a^ (yes/n+/-)
**1**	43	F	9600	0.06	1	no	no	no	no	no
**2**	79	M	6200	0.03	2	no	yes	yes	no	ye
**3**	69	M	6900	0.03	2	yes	yes	yes	no	yes
4	62	M	6000	0.13	2	yes	no	yes	yes	yes
**5**	75	M	11300	0.64	2	no	yes	yes	no	yes
**6**	60	F	12400	3.53	2	no	no	no	ye	yes

F: female, M: male, WBC: white blood cell count, CRP: c-reactive protein, AC: acute cholecystitis (grade 1–3), PTGBD: Percutaneous transhepatic gallbladder drainage, PTGBA: Percutaneous transhepatic gallbladder aspiration, EL：endoscopic lithotripsy.

a Preoperative MRCP findings were including detection of the cystic duct (yes/no) and GB (yes/no).

**Table 2:** Operational and postoperative outcomes.

case	DAO	OT (minutes)	Bleeding (g)	POBL (yes/no)	LHS (days)	Post MRCP
1	1	182	1	no	6	–
2	1	134	1	no	5	–
3	10	133	1	no	7	NP
4	1	199	100	no	6	–
5	1	138	50	no	7	NP
6	13	112	1	no	6	NP

DAO: day from admission to operation, OT: operating time, POBL: post-operative bile leakage, LHS: length of hospital stay, Post MRCP: Post-operative MRCP findings, NP: nothing particular.

## 4 Discussion

Routine LC has been established as a common surgical procedure for benign GB diseases. TG18 recommends emergency LC for patients with acute cholecystitis (AC) within 72 h of onset with stable general condition, therefore emergency LC cases may increase in the near future. However, not all medical centers are able to perform emergency surgery for AC due to their systemic limitations and availability of anesthesiologists and surgeons. So, several medical centers, including our center, performed conservative treatment (72 h or more) before LC. In addition, after conservative treatment, such as PTBD or antibiotic administration etc., severe inflammation and fibrosis is commonly seen around GB, and as a result becomes very difficult to identify CVS at the time of LC. If LC is continued to be implemented in such difficult cases, it may induce serious complications such as vaso-biliary injury (VBI), bowel injury, and intraoperative bleeding. Therefore, Strasberg et al. proposed subtotal cholecystectomy, which involves partial resection of the GB., The procedure has been implemented in various facilities thus far, and its effectiveness in prevention of VBI has been well proven.

On the other hand, LSC has some outstanding complications such as postope. BL, remnant of GB stone, and residual GB cancer. In particular, postope BL appears in early postoperative days and requires immediate treatment. Few reports describe various closing techniques for remnant of GB, among which some effectively prevented postope. BL.

Giraldo et al. described that a classification of subtotal cholecystectomy, and several closing techniques for remaining gallbladder stump for prevention of bile leakage [4]. The techniques of remnant of GB closure after LSC had been machine suture and manual hand suture. Usually, remnant of GB is very fragile, and conventional techniques have sometimes resulted in inadequate closure or incomplete suturing due to wall rupture. Some of authors described new closing techniques for remnant of GB. Matsui et al. reported that suture closing for remnant of GB with omentum plugging was more effective in preventing postope BL rather than simple suture closing of the cystic duct (unknown under laparoscopic) [8]. Choi et al. have proposed a method of covering and suturing the falciform ligament to wall of the remnant of GB without suturing technique [9]. Fujiwara et al. performed suture closure of remnant of GB using barbed sutures in 2 cases [10].

In our department, if the patients require suture closing of remnant of GB, we converted to laparotomy at the beginning of subtotal cholecystectomy under LC. From 2018, we tried to perform suture closing of remnant of GB under laparoscopy, however in the first case, we performed simple interrupted suture closing, and the patient developed postope. BL at 1st POD. After that experience, we modified our procedure as in this present study.

The LapraTy® suture clip did not require any ligation, and it was able to adjust the tightening without loosening the tension for thickened and/or fragile remnant of GB tissues. Furthermore, it was possible to perform very effective suture on GB tissues without any tension and damage. In addition, the suture is covered with an omentum and was able to prevent bile leakage. In LSC cases, there is also a risk of developing residual gallstones or cholecystitis postoperatively, but in our new technique of suturing the remnant of GB after confirming bile outflow (provided there is no stone present in the remnant of GB), it can be argued that the technique also reduces the risk of postoperative residual gallstones.

In this short-term result, we never experienced postope. BL in the accrued patient group, and the technique was a very useful method for preventing postope. BL. Furthermore, we did not have any cases of residual gallstones, cholecystitis, and abscess formation using this technique so far.

On the other hand, it is undeniable that increase in LSC cases leads to increase in the risk of malignancy. We suggest a large follow-up study to be conducted in the future.

In best of our knowledge, this is the only report that describes the efficacy of using the covering of momentum patch to the remnant of GB. There are several limitations to this study. First, the small number of patients in this study may have affected the validity of the results. Second, significant selection bias existed because of the retrospective nature of this study. A prospective analysis with larger patient accrual should be conducted to address this limitation. The effectiveness of the tissue coverage needs to be continuously examined through various treatment experiences. This study is a proposal for a new surgical technique, and we plan to increase the number of cases in the future to continuously verify the presence or absence of complications.

## 5 Conclusion

Closure with continuous sutures using LapraTy® suture clips with omental covering during LSC was useful technique for remnant of GB. closing.

## Ethics approval and consent to participate

This study was approved by the Research and Ethics Committee of Tokyo Medical University (study approval no.: T2020–0352 and T2022-0149). This retrospective study was conducted in compliance with all regulations. In addition, informed consent was obtained from all patients. This procedure performed in studies involving human participants were in accordance with the ethical standards of the institutional research committee and with the 1995 Helsinki Declaration.

## Author contribution statement

Mitsugi Shimoda: Conceived and designed the experiments; Analyzed and interpreted the data; Contributed reagents, materials, analysis tools or data; Wrote the paper.

Yu Kuboyama: Performed the experiments; Analyzed and interpreted the data; Wrote the paper.

Shuji Suzuk: Analyzed and interpreted the data.

## Data availability statement

The authors do not have permission to share data.

## Declaration of competing interest

The authors declare that they have no known competing financial interests or personal relationships that could have appeared to influence the work reported in this paper.
