# Supplementary material for: Continuous suture closure using a LapraTy® suture clips is an effective method for reconstruction of cystic duct stump after laparoscopic subtotal cholecystectomy
Source: Heliyon. 2023 Sep 21;9(9):e20043. doi: 10.1016/j.heliyon.2023.e20043 (PMC10559765; doi:10.1016/j.heliyon.2023.e20043)
Supplement: Multimedia component 1 [file mmc1.pptx]

## Slide 1
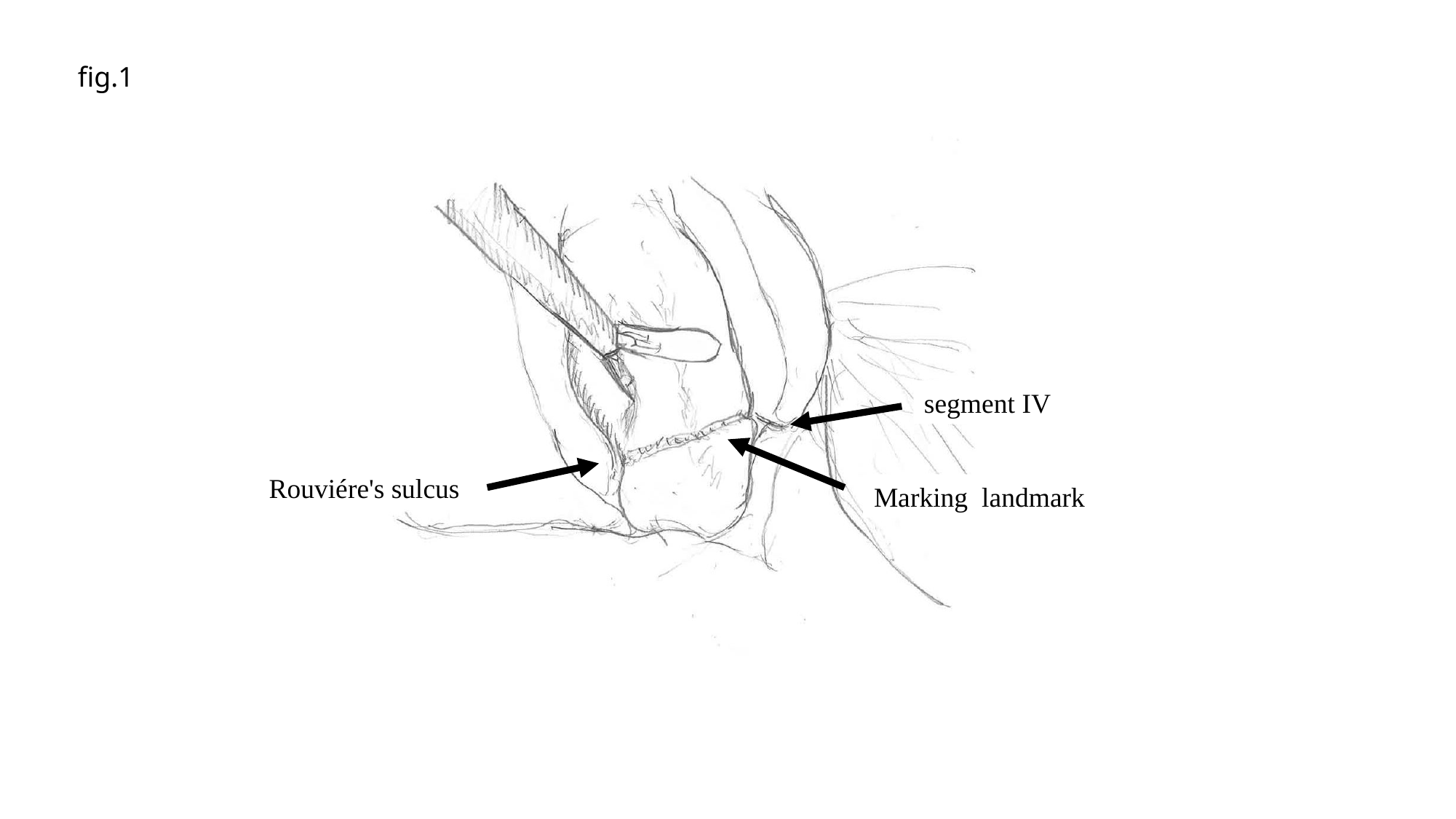

fig.1
segment IV
Rouviére's sulcus
Marking landmark

## Slide 2
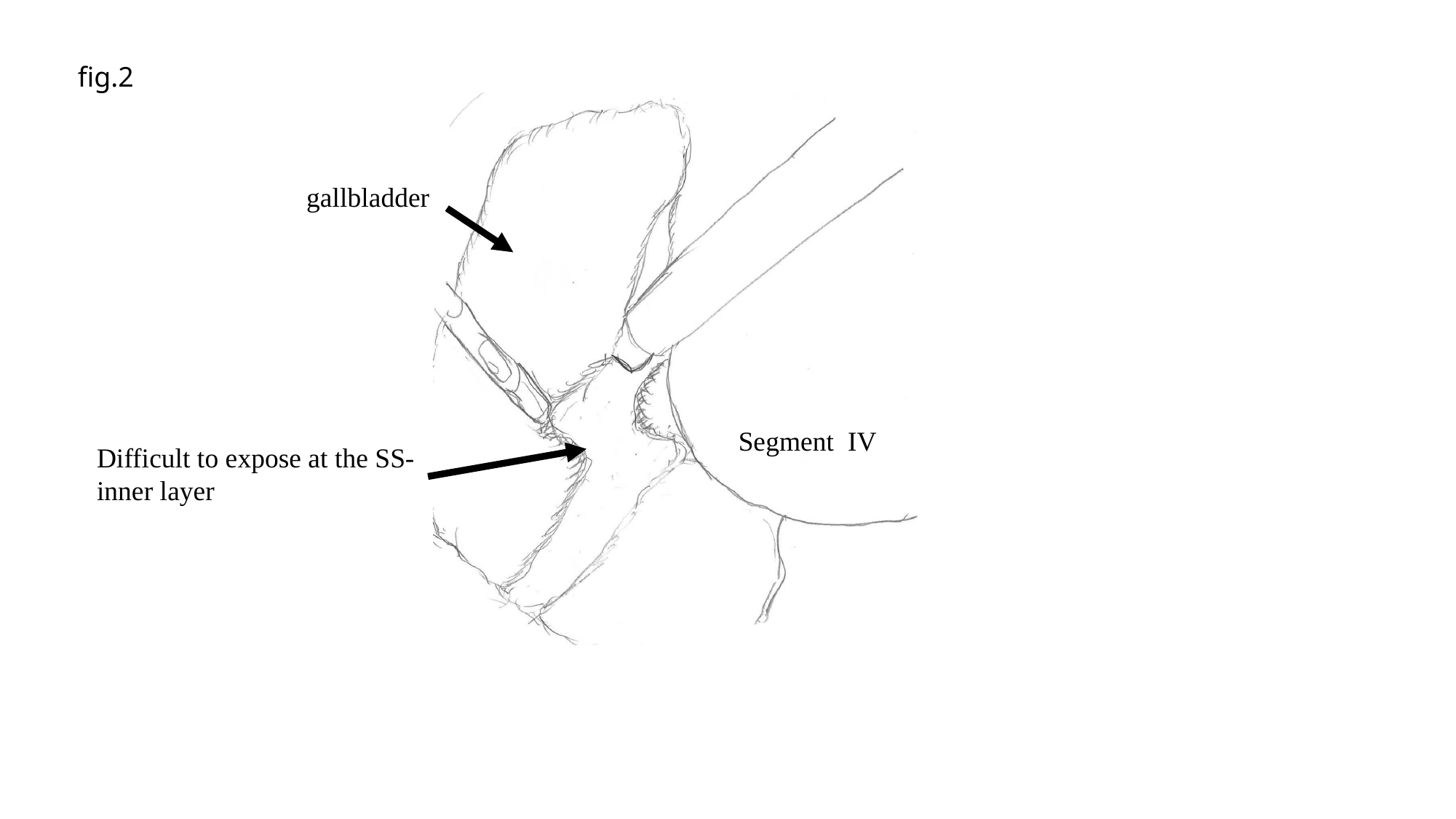

fig.2
gallbladder
Segment IV
Difficult to expose at the SS-inner layer

## Slide 3
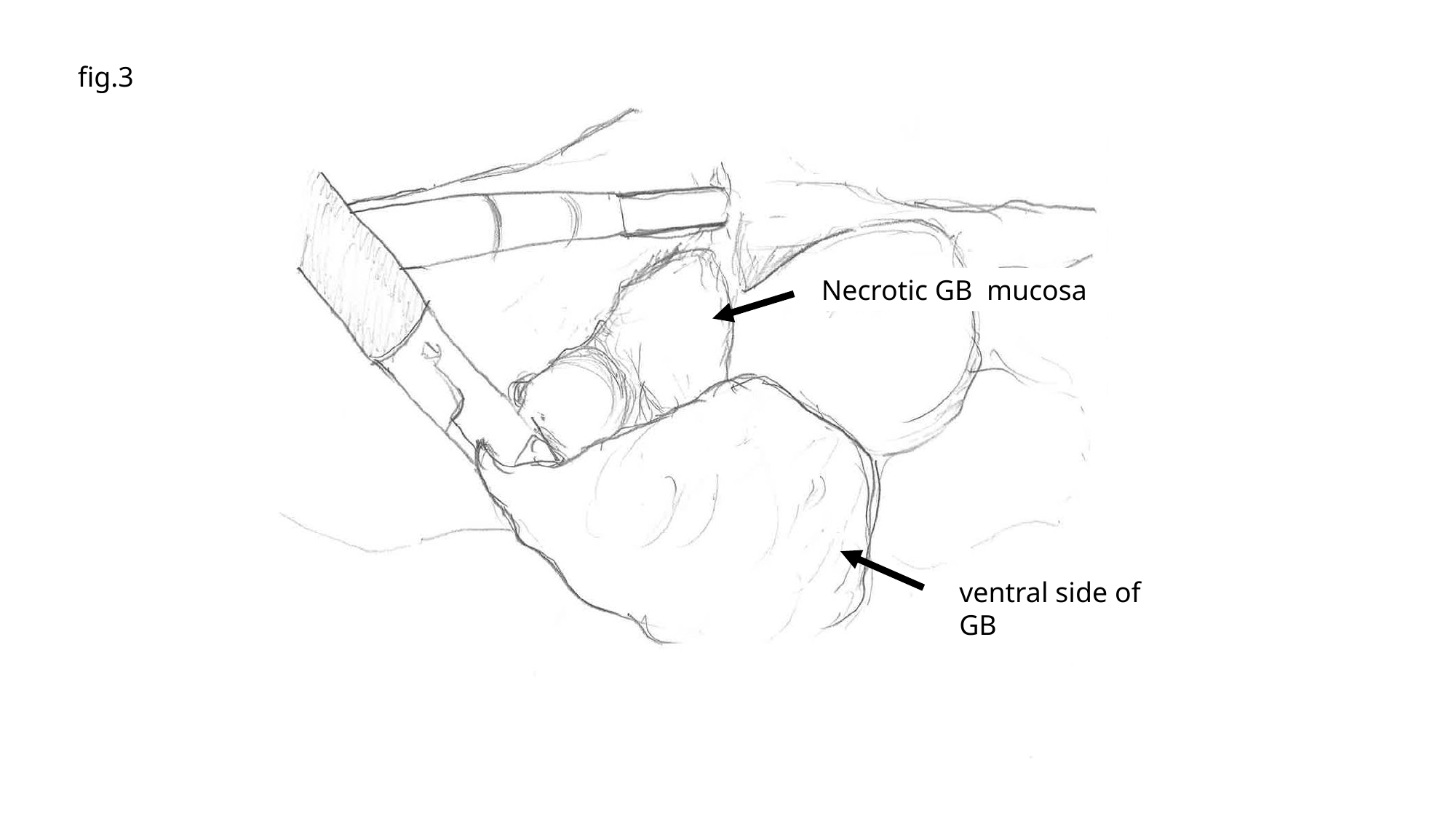

fig.3
Necrotic GB mucosa
ventral side of GB

## Slide 4
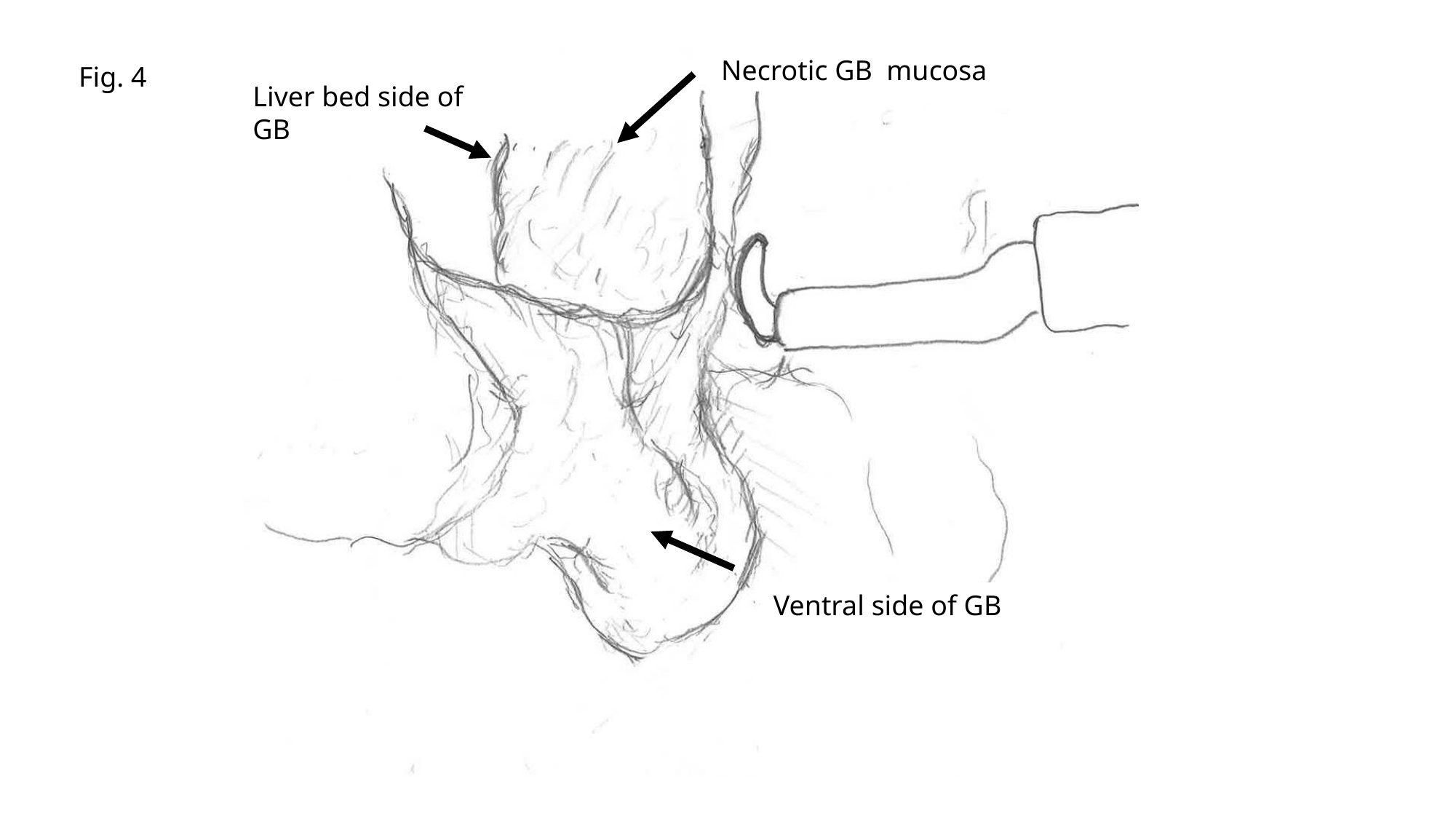

Necrotic GB mucosa
Fig. 4
Liver bed side of GB
Ventral side of GB

## Slide 5
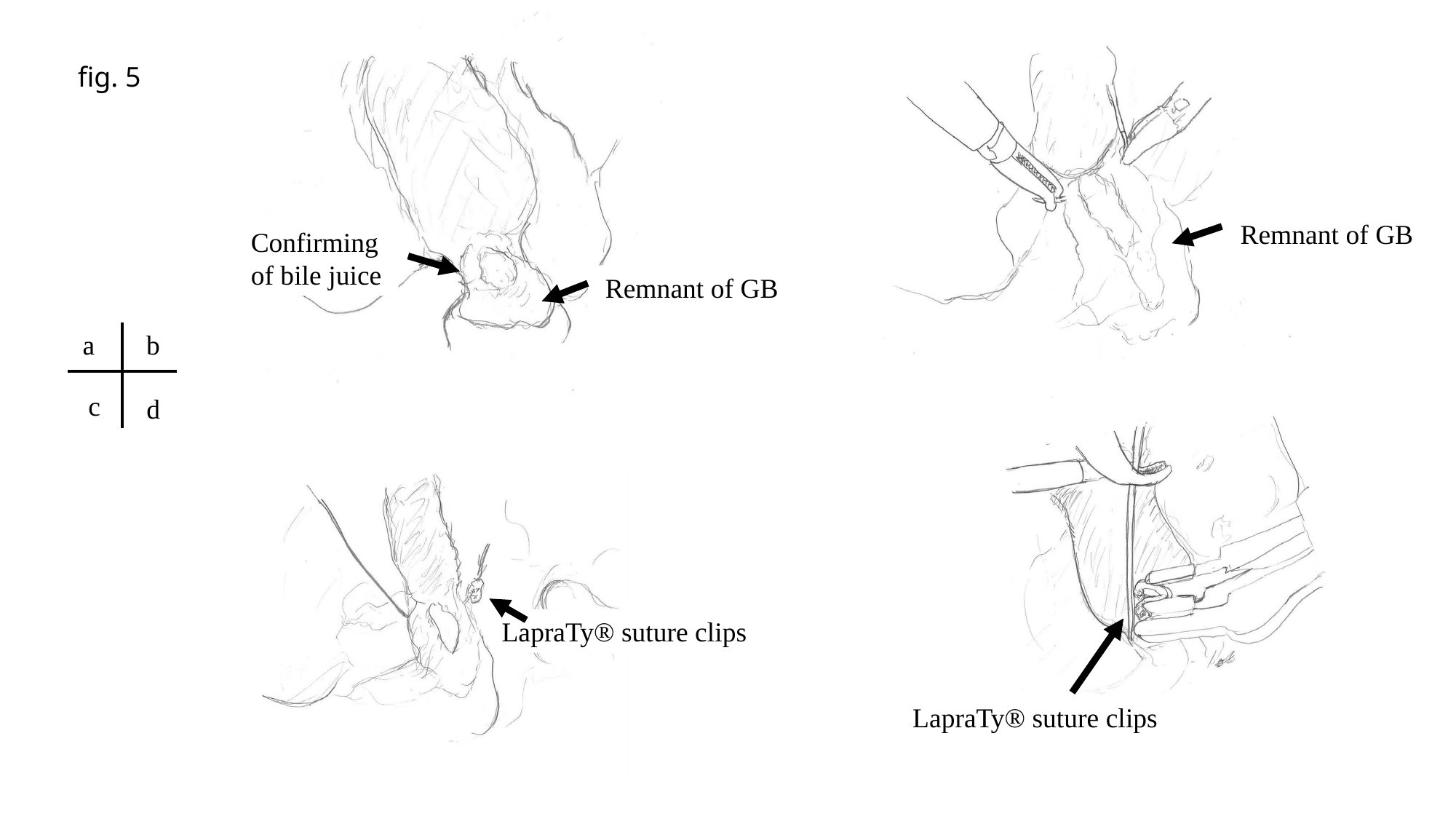

Confirming of bile juice
 Remnant of GB
 Remnant of GB
fig. 5
a
LapraTy® suture clips
b
c
d
LapraTy® suture clips

## Slide 6
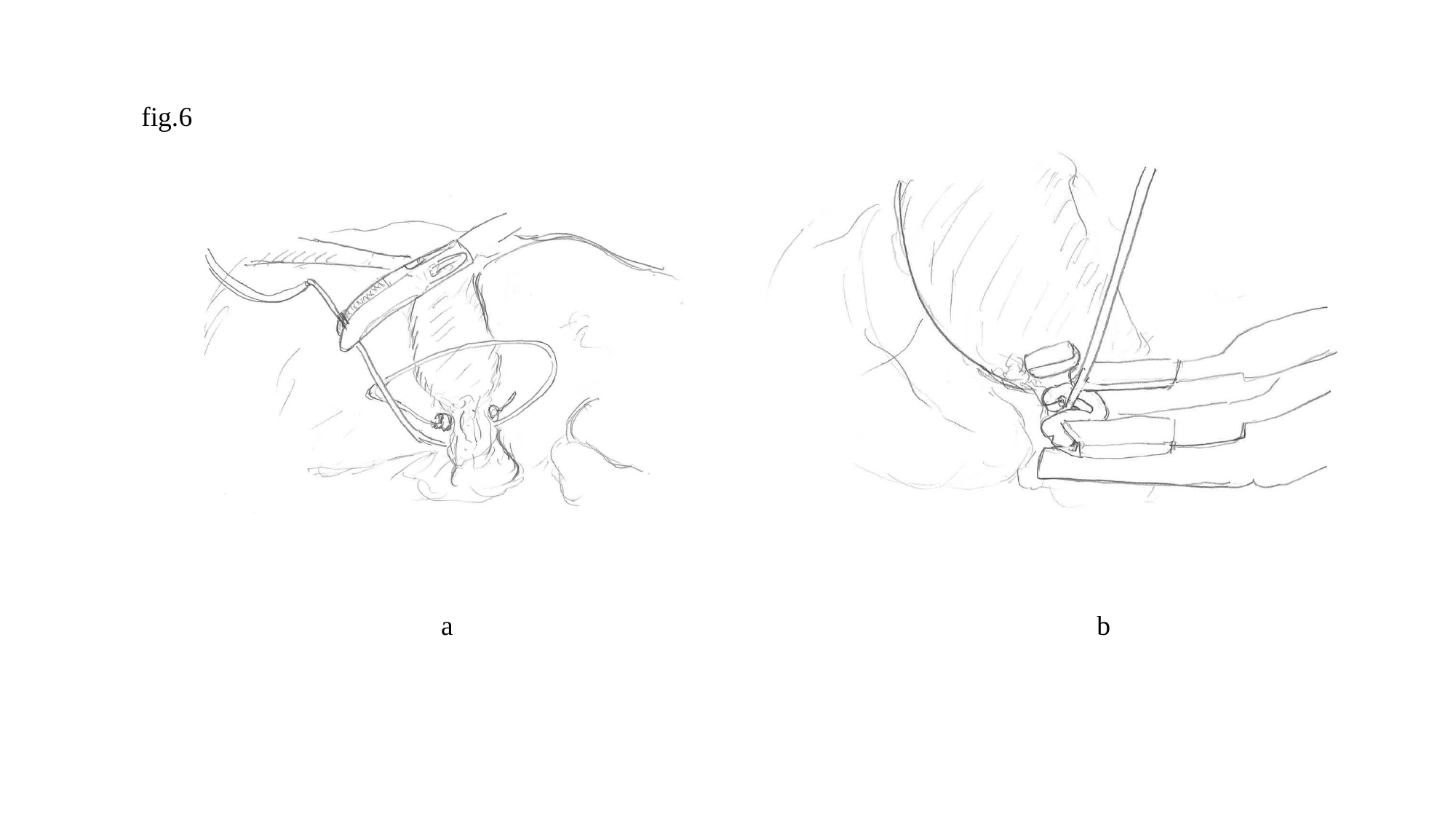

fig.6
a
b

## Slide 7
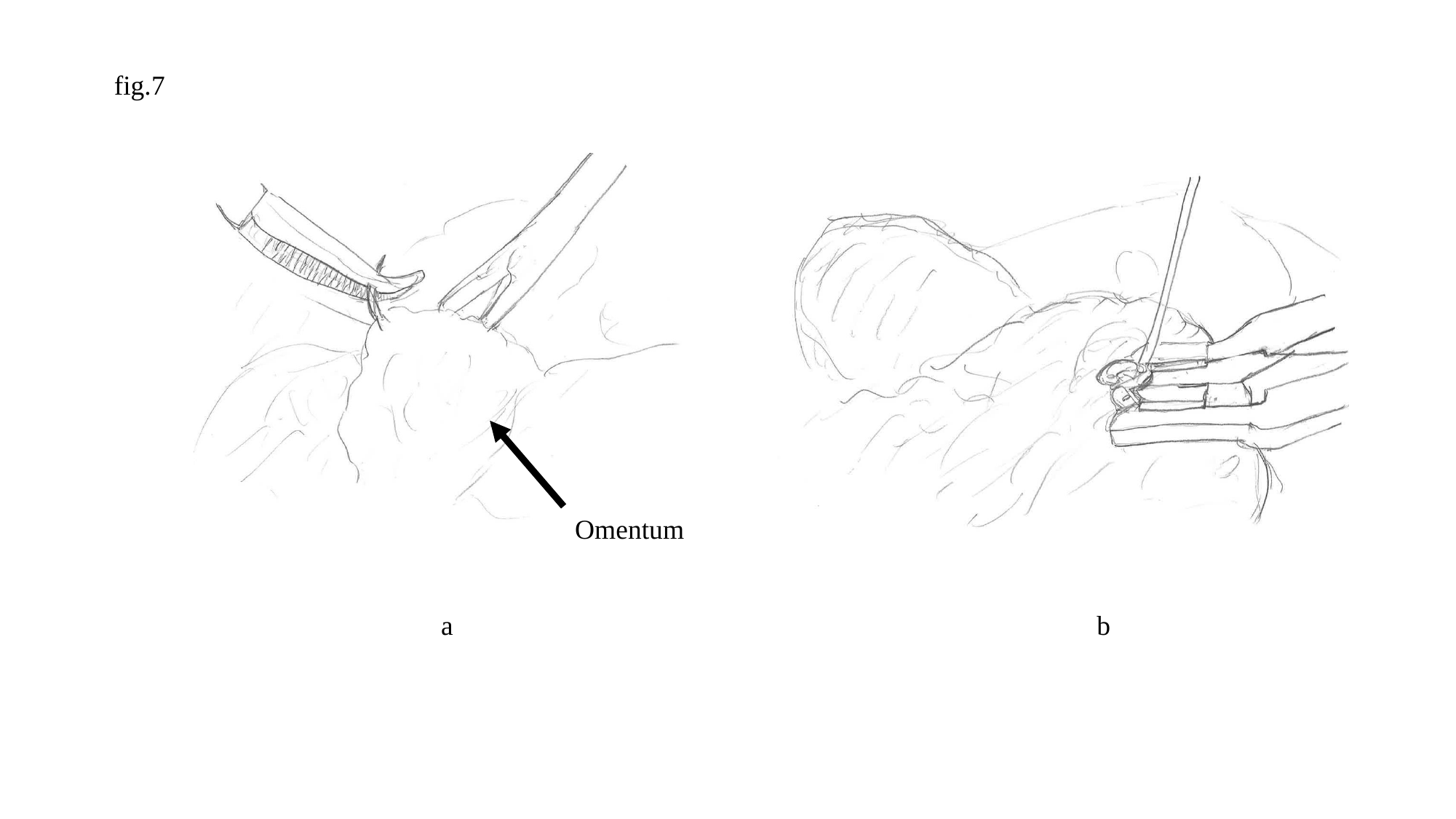

fig.7
Omentum
a
b
